# A Design of Quad-Element Dual-Band MIMO Antenna for 5G Application

**DOI:** 10.3390/mi14071316

**Published:** 2023-06-27

**Authors:** Chengxin Shi, Zhuolin Zhao, Chengzhu Du

**Affiliations:** 1 College of Electronics and Information Engineering, Shanghai University of Electric Power, Shanghai 200090, China; 2 State Grid East China Power Grid Shanghai Municipal Electric Power Company, Shanghai 200002, China

**Keywords:** dual-band, quad-element, MIMO, CPW, 5G communication, high isolation

## Abstract

A dual-band four-element MIMO antenna was designed and fabricated with enhanced isolation. The introduced antenna was fed by a coplanar waveguide (CPW) and consisted of four identical monopole antenna elements placed perpendicular to each other. A cross-shaped stub and orthogonal placement of four elements were introduced for high isolation. Modified ground structure was used for extending bandwidths. The measured results demonstrate that the introduced antenna has double bands (S11 < −10 dB) covering 3.28–4.15 GHz and 4.69–6.01 GHz, with fractional bandwidths of 23.4% and 24.7% and a high isolation S21, S31 better than 19 dB. The curves of the envelope correlation coefficient (ECC) and diversity gain (DG) were less than 0.0025 and higher than 9.999, respectively, indicating a low correlation between antenna elements. Furthermore, gain, efficiency, channel capacity loss (CCL), total active reflection coefficient (TARC) and mean effective gain (MEG) have all been investigated over the operating band to determine the antenna’s diversity performance. In accordance with the simulated and measured results, it confirms that the proposed antenna is appropriate for 5G applications.

## 1. Introduction

As the requirement for mobile communication increases, the fifth-generation mobile communication technology(5G) has emerged with faster speeds and more reliable capabilities. Among 5G communication, the 3.45 GHz and 4.9 GHz bands have drawn great attention as they are allocated within sub-6 GHz bands. At the same time, MIMO technology has attracted attention for its advantages of the rise of channel capacity and improvement of multipath fading effects. So, 5G communication combined with MIMO technology will further improve the transmission rate and efficiency in comparison to the single antenna system.

During the design process of many proposed MIMO antennae [1,2,3,4,5,6,7,8,9,10,11,12,13,14,15,16,17,18,19], there exists a complex problem of maintaining the compact volume and achieving high isolation at the same time. Many tools have been used to decrease the mutual coupling, including defected ground structure (DGS), parasitic element, neutralization line, passive resonator, electromagnetic bandgap (EBG) and so on. Antennae proposed in [1,2,3] use the method of the passive resonator. In [1], four split-ring resonators rowed clockwise were placed in the center of the substrate to enhance decoupling capability. In [2], monopole antenna elements loaded with split ring resonators can not only produce extra resonant frequency but can also act as a reflection to reduce the coupling current. In [3], a near-field resonator was used as a decoupling structure to achieve a high isolation better than 20 dB. The neutralization line as a standard technique has also been used in [4,5,6]. In [4], a wideband neutralization line with a circular disc in it is connected and inserted between the antenna elements for high isolation. In [5], the two-port antenna array was constructed using a neutralization line, resulting in the array port isolation of up to 30 dB. By introducing defected ground structure (DGS), the mutual coupling has been increased effectively in [6,7].

Previously, many dual-band MIMO antennae with two elements or four elements have been designed. A two-port antenna with double bands is presented in [12], exhibiting better than 15 dB isolation. Two microstrip-fed two-element two-band antennae for 5G applications are presented [15,17], the isolation of the antenna in [15] is better than 15 dB, and the other isolation of the antenna in [17] is better than 16 dB. Dual-band MIMO antennae with four elements are proposed in both [18,19]. A four-element antenna with a miniaturized design and dual-band characteristics has been presented in [18], which exhibits an isolation value of more than 18.4 dB. In [19], a microstrip-fed quad-port dual-band antenna is designed with an isolation better than 19 dB. However, the impedance bandwidths of working bands are narrow in these references on four-element MIMO antennae.

In this article, a quad-element antenna fed by CPW with the characteristic of double bands is designed for 5G systems. The MIMO antenna is printed on an FR4 substrate, operating in 5G bandwidths covering 3.3–3.6 GHz and 4.8–5.0 GHz, achieved by two inverted L-shaped monopoles. Additionally, it is comprised of four monopole antenna elements. A cross-shaped stub and orthogonal placement of four elements are introduced for high isolation of the MIMO antenna. Furthermore, the ground structure is modified to extend the bandwidth across the 5G operation bands. A detailed description of the evolution of antenna design, S-parameters, radiation patterns and diversity performance, including gain, efficiency, ECC, DG, CCL, MEG and TARC follows in Section 2, Section 3 and Section 4.

## 2. Geometry and Design of Antenna

### 2.1. Antenna Geometry

**Figure 1:**
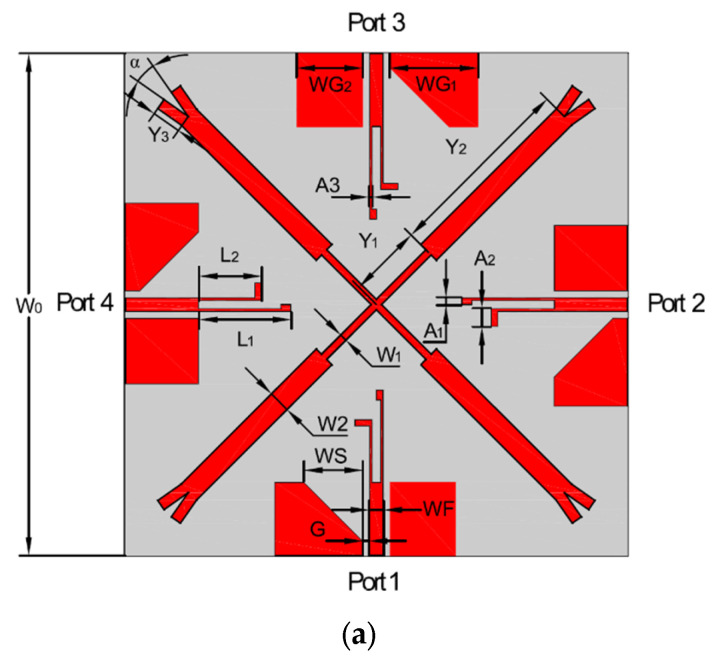

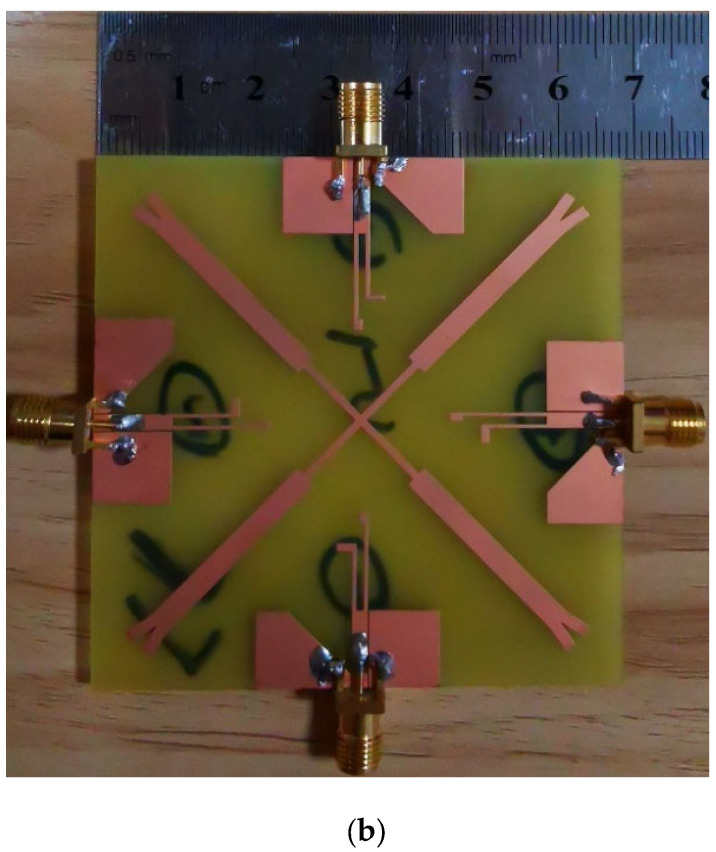
Geometry and photograph of the MIMO antenna. (**a**) Antenna structure, (**b**) manufactured antenna.

The presented MIMO antenna, fed by a 50-Ω microstrip of coplanar waveguide (CPW) line, is printed on a 0.8 mm FR4 substrate with an overall dimension of 68*68*0.8 mm^3^, a relative permittivity of 4.4 and a loss tangent of 0.02. It consists of four elements with two inverted L-shaped monopoles to achieve two operation bands (3.3–3.6 GHz and 4.8–5.0 GHz). The design and optimization processes are implemented by Ansoft HFSS 13.0. The configuration and the photograph of the introduced antenna are shown in Figure 1 with the dimensions (in mm): G = 0.2, WF = 2, W0 = 68, LG = 10, WG1 = 12, WG2 = 9, WS = 8, L1 = 12.5, L2 = 8.5, A1 = 0.6, A2 = 2,A3 = 0.4, Y1 = 10, Y2 = 16, Y3 = 4, W1 = 1, W2 = 3, α = 20 deg.

Due to the symmetry of the arrangement of these four antenna elements, the conclusion of S_12_ = S_23_ = S_34_ = S_41_ and S_31_ = S_42_ can be obtained. To facilitate better analysis, the simulated and tested S-parameters are presented as follows in the paper.

### 2.2. Design Process

**Figure 2:**
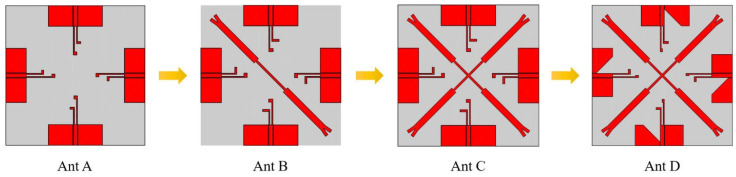
Antenna design process.

**Figure 3:**
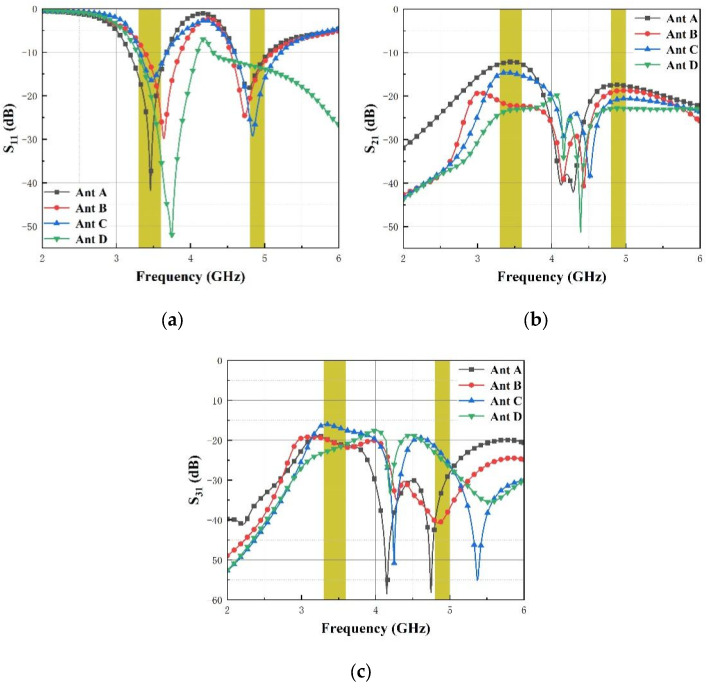
Comparison of S-parameters for Ant A, Ant B, Ant C and Ant D. (**a**) S_11_, (**b**) S_21_ and (**c**) S_31_.

A diagram of the antenna’s design process is described in Figure 2, starting from Ant A to Ant D. The simulated S-parameters of S_11_, S_21_ and S_31_ of each antenna are presented in Figure 3.

The design originated from the monopole antenna with two inverted L-shaped stubs of λ/4 which can operate within 3.3–3.6 GHz and 4.8–5.0 GHz. There are four steps for MIMO antenna design. In the first step, the original MIMO antenna denoted as Ant A consists of four antenna elements arranged perpendicularly, located on the four sides of the substrate, the MIMO antenna with orthogonal placement of antenna elements has a higher isolation than the antenna with parallel placement. In the second step, Ant B is designed by adding a double-Y-shaped stub on Ant A for high isolation. In the third step, Ant C is designed by adding another double-Y-shaped stub on Ant B for higher isolation. In the fourth step, Ant D is designed by cutting partial ground on Ant C, partial ground is introduced for improving the bandwidth. Figure 3 shows S-parameters for Ant A, Ant B, Ant C and Ant D. It can be seen that the introduction of the isolator causes the resonant frequencies shift, but the bandwidth (S_11_ < −10 dB) has been extended obviously in Ant D compared to other antennae. In Figure 3b, the S_21_ of Ant A is around −12 dB within the operating bands firstly. To decrease coupling among antenna elements, a double-Y-shaped stub is arranged among antenna elements in Ant B first, then another double-Y-shaped stub is placed among antenna elements in Ant C. The cross-shaped stub acts as a reflector to eliminate the coupling of electromagnetic energy in space. In Figure 3c, the maximum isolation (S_31_) of Ant D has been increased by 5 dB compared to Ant C at the first operating band. Finally, the values of S_21_ and S_31_ are all less than −20 dB. 

### 2.3. Parametric Study

**Figure 4:**
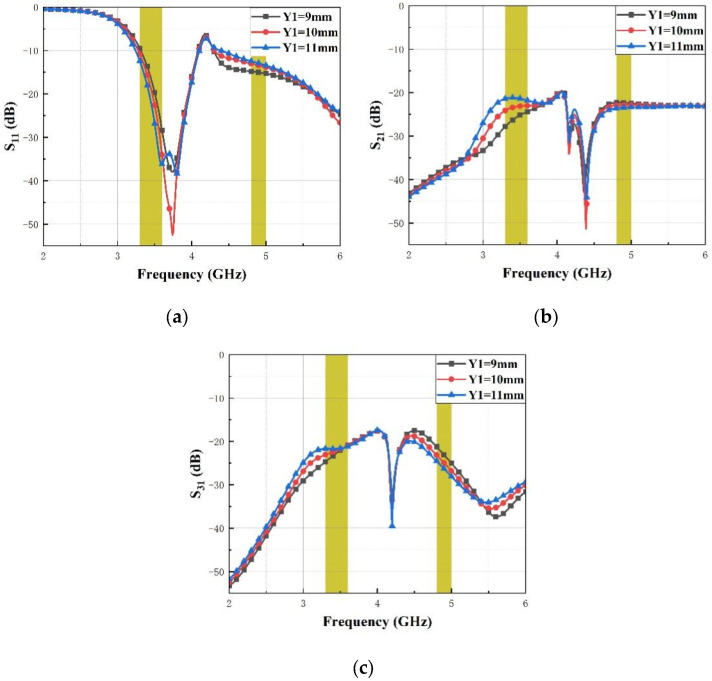
The proposed MIMO antenna simulated S-parameters for various Y1 (**a**) S_11_, (**b**) S_21_ and (**c**) S_31_.

**Figure 5:**
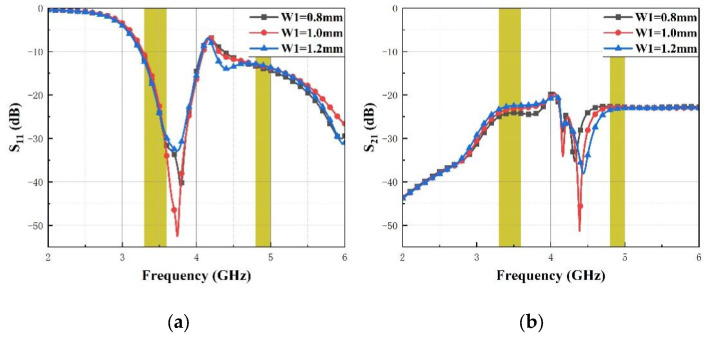

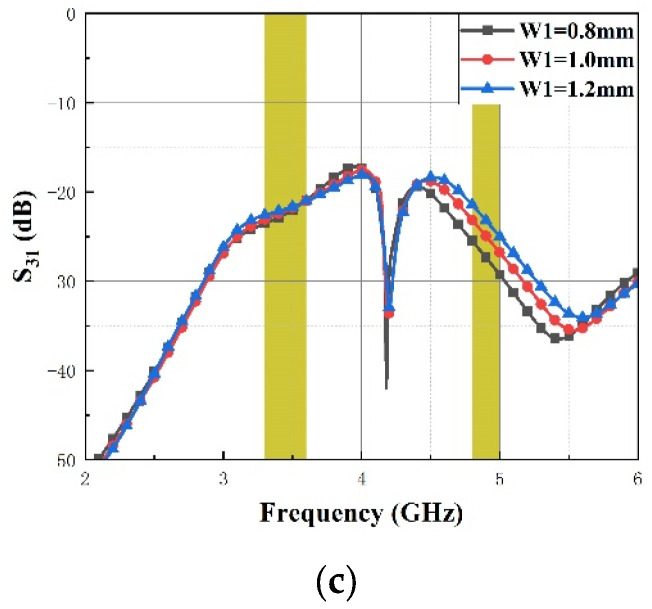
The proposed MIMO antenna simulated S-parameters for various W1 (**a**) S_11_, (**b**) S_21_ and (**c**) S_31_.

Figure 4 shows the impact of the thin stub length Y1 on S_11_, S_21_ and S_31_. It can be seen that with the increase in the length of the stub Y1 from 9 mm to 11 mm, the impedance bandwidth (S_11_ < −10 dB) at the lower band gets wider and the upper band isolation (S_31_) increases gently, while the isolation (S_21_) decreases obviously at the lower band. Figure 5 presents the impact of the thin stub width W1 on S_11_, S_21_ and S_31_. It is shown that with the increase in the width of the thin stub W1 from 0.8 mm to 1.2 mm, the impedance bandwidth (S_11_ < −10 dB) hardly changes at two operating bands and the lower band isolation (S_21_) decreases gently, while the isolation (S_31_) decreases obviously at the upper band. 

### 2.4. Current Distribution

**Figure 6:**
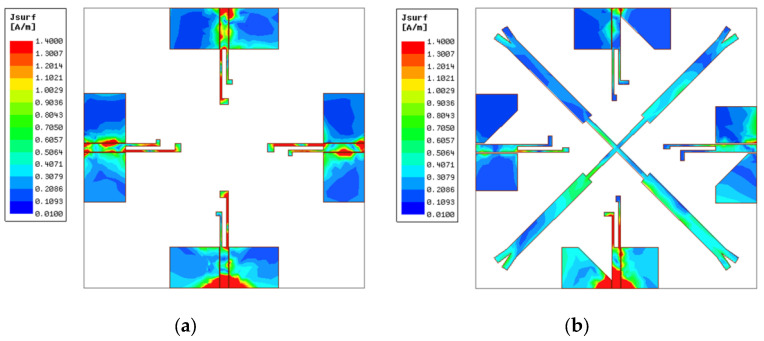
Current distributions at 3.45 GHz. (**a**) Ant A and (**b**) Ant D.

**Figure 7:**
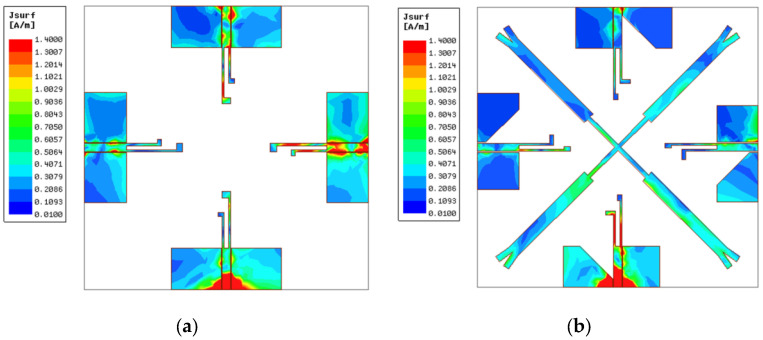
Current distributions at 4.9 GHz. (**a**) Ant A and (**b**) Ant D.

To further comprehend the electromagnetic mechanism of decoupling structure, current distributions of Ant A and Ant D at 3.45 GHz and 4.9 GHz were simulated and are illustrated in Figure 6 and Figure 7. In Figure 6a, it can be observed that when there is no decoupling structure between antenna elements, port 1 delivers a large amount of current to other ports at 3.45 GHz, leading to poor performance of isolation. While in Figure 6b the coupling current is significantly decreased after introducing the crosswise stub and transforming the structure of the ground plane, it turns out that the crosswise stub can block the current flowing from port 1 to other ports to a certain degree. In addition, by reducing the width of the ground, the current path is extended. Consequently, coupling current is prevented from flowing to other elements to destroy the operation of antenna, leading to the achievement of an enhanced isolation better than 20 dB. Ant A and Ant D current distributions at 4.9 GHz are illustrated in Figure 7. It reveals that the cross-shaped stub with the combination of the modified ground plane has a significant effect and decreases electromagnetic energy coupling, making the mutual current diminished at the upper band.

## 3. Experiment Verification

### 3.1. S-Parameter

**Figure 8:**
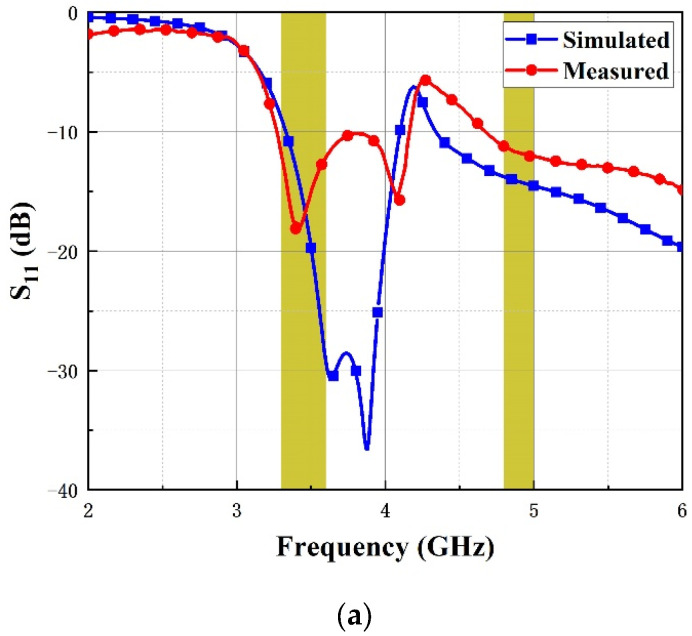

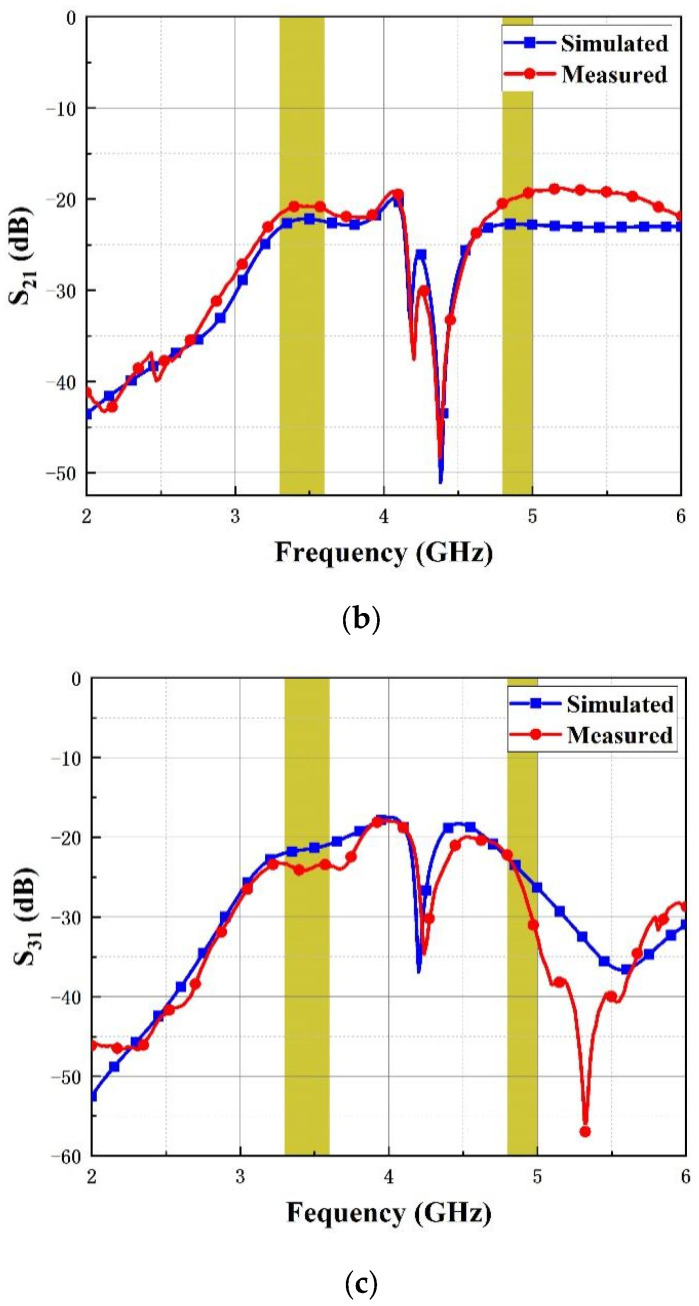
Comparison of S-parameters. (**a**) S_11_, (**b**) S_21_ and (**c**) S_31_.

To verify and inspect the scheme above, the two-band MIMO antenna was fabricated and the performance of it was tested by N5230A PNA-L Network Analyzer. Figure 8 describes the measured curves of S-parameters including S_11_, S_21_ and S_31_ in comparison to simulated ones. The measured impedance bandwidths range from 3.28–4.15 GHz and 4.69–6.01 GHz with a fractional bandwidth of 23.4% and 24.7%, respectively. The isolation generally reached to more than 20 dB at the lower band while the upper band also had a desirable isolation with a coupling coefficient better than 19 dB. In terms of the comparison of the simulation and tested curves, there is still a tiny distinction between them. The difference is majorly attributed to the SMA connector loss and manufacturing error, but it is within permissible range.

### 3.2. Radiation Patterns

**Figure 9:**
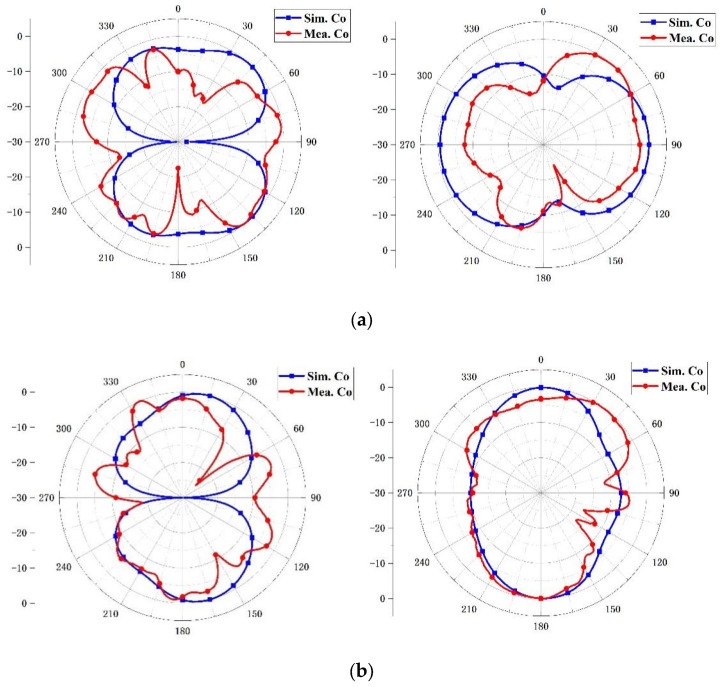
Radiation patterns at (**a**) 3.45 GHz and (**b**) 4.9 GHz.

Simulation and measurement of radiation patterns in the E-plane and H-plane are performed to determine the radiation mechanism for the illustrated MIMO antenna. Due to the symmetrical arrangement, only the case where Port 1 is excited and the others are matched load has been tested. The normalized results have been plotted in Figure 9. The diagram demonstrates that the simulated and measured results of coplanar polarization appear to be approximately omnidirectional—although there exists a little difference—and the reason for this is that the antenna is measured in the 2-D microwave anechoic chamber.

## 4. Diversity Performance

### 4.1. Gain and Efficiency

**Figure 10:**
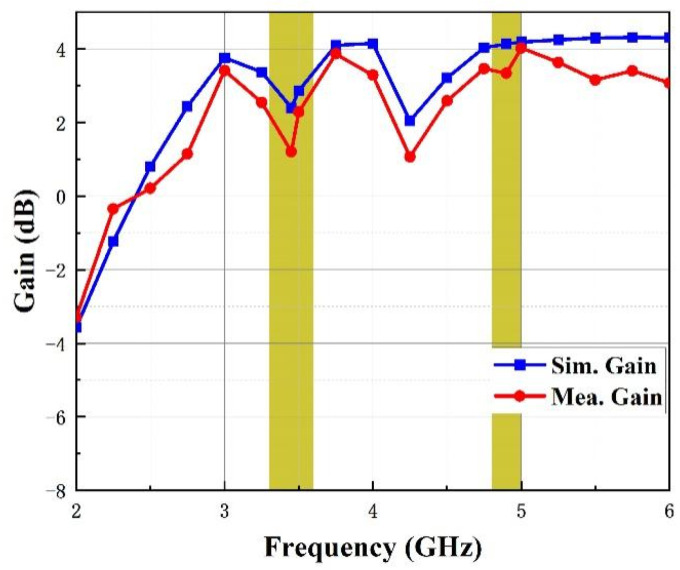
Gain of the antenna.

A comparison of simulated and tested peak gain curves is shown in Figure 10. Among the results, the simulated gain is around 2.5 dB and 4 dB at the lower and upper band, respectively, while the measured gain is around 1.2 dB over the operating bands. The simulated and tested results differ slightly, which can generally be attributed to the interference of the Microwave Anechoic Chamber and SMA connector loss.

**Figure 11:**
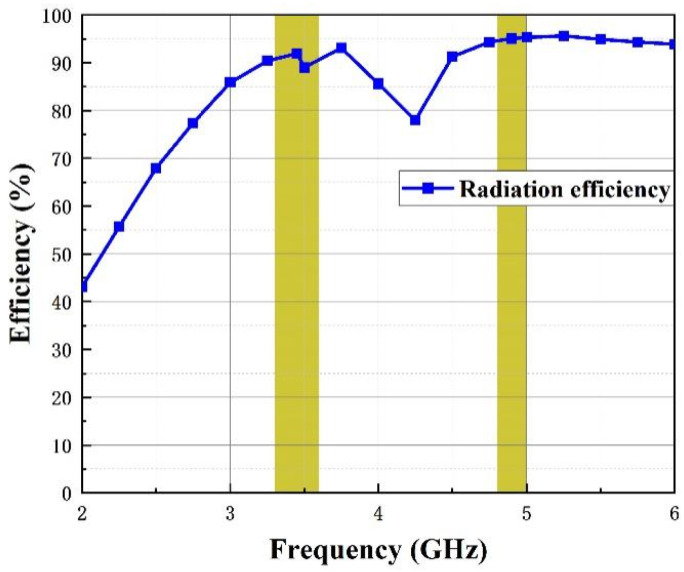
Efficiency of the antenna.

The efficiency can also help to describe the antenna’s performance. It can be observed from Figure 11 that radiation efficiency is generally better than 90% over the two operating bands.

### 4.2. ECC and DG

As a valuable parameter, the envelope correlation coefficient (ECC) is used in MIMO system to evaluate the extent of correlation between antenna elements. It can be calculated by following expression [20]:

(1) ρei,j,N=∑n=1NSi,n∗Sn,j∏k=i,j1−∑n=1NSk,n∗Sn,k122

where i and j denote the serial number of antenna ports and N is the total number of ports. Based on the equation above, we can obtain the value of ECC_12_ (ρ_e_(1,2,4)) and ECC_13_ (ρ_e_(1,3,4)) by the following equation:

(2) $$\rho_{e}\left(1,2,4\right)=\frac{{\left|S_{11}^{*}S_{12}+S_{21}^{*}S_{22}+S_{13}^{*}S_{32}+S_{14}^{*}S_{42}\right|}^{2}}{\left(1-{\left|S_{11}\right|}^{2}-{\left|S_{21}\right|}^{2}-{\left|S_{31}\right|}^{2}-{\left|S_{41}\right|}^{2}\right)\left(1-{\left|S_{12}\right|}^{2}-{\left|S_{22}\right|}^{2}-{\left|S_{32}\right|}^{2}-{\left|S_{42}\right|}^{2}\right)}$$

(3) $$\rho_{e}\left(1,3,4\right)=\frac{{\left|S_{11}^{*}S_{13}+S_{12}^{*}S_{23}+S_{13}^{*}S_{33}+S_{14}^{*}S_{43}\right|}^{2}}{\left(1-{\left|S_{11}\right|}^{2}-{\left|S_{21}\right|}^{2}-{\left|S_{31}\right|}^{2}-{\left|S_{41}\right|}^{2}\right)\left(1-{\left|S_{13}\right|}^{2}-{\left|S_{23}\right|}^{2}-{\left|S_{33}\right|}^{2}-{\left|S_{43}\right|}^{2}\right)}$$

As an additional index to evaluate MIMO antenna performance, diversity gain (DG) can be calculated using following equation:

(4) $$\mathrm{DG}=10\sqrt{1-{\left(\mathrm{ECC}\right)}^{2}}$$

**Figure 12:**
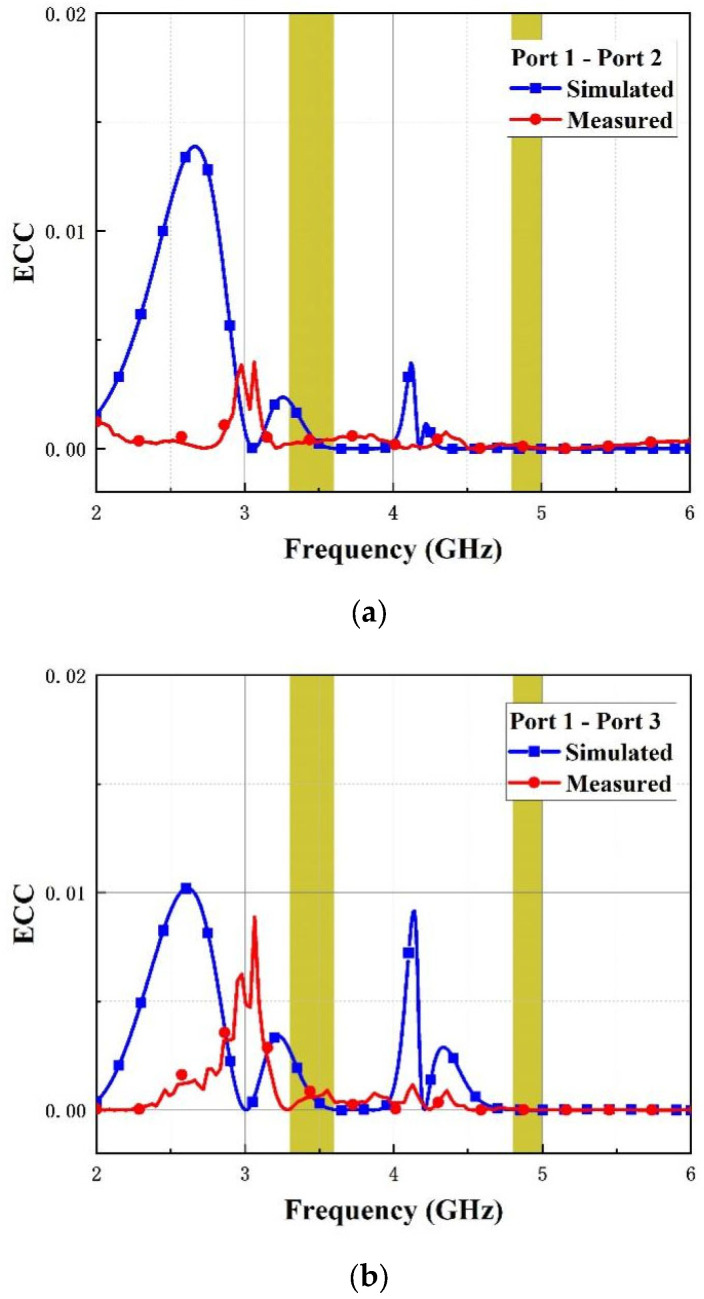
ECC of the introduced antenna. (**a**) Port1-port2 and (**b**) port1-port3.

**Figure 13:**
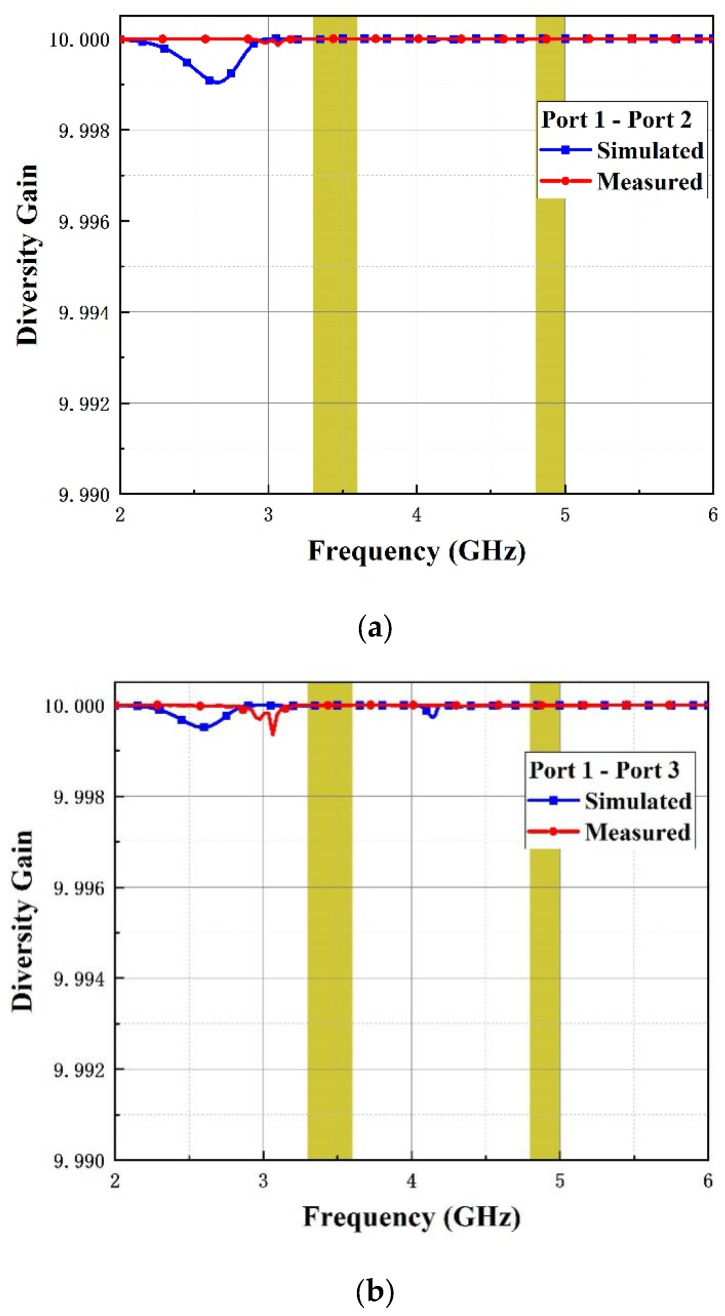
DG of the introduced antenna. (**a**) Port1-port2 and (**b**) port1-port3.

Figure 12 and Figure 13 show the simulated and measured curves of ECC and DG among antenna elements (Ant 1, Ant 2) and (Ant1, Ant 3), respectively. As is illustrated in the figures, the value of ECC is below 0.0025 and DG exceeds 0.999 over the operating bands, which proves that the MIMO antenna possesses good performance.

### 4.3. CCL and MEG

Within a communication system, the channel capacity loss (CCL) refers to the achievable upper limit of transmission speed. The practically acceptable CCL is below 0.4 bits/s/Hz within working bands. For a four-port antenna, it can be calculated as follows [20]:

(5) $$\mathrm{CCL}=-\log_{2}\det\left(\alpha^{R}\right)$$

where $\alpha^{R}=\left[\begin{array}{cc} \begin{array}{cc} \alpha_{11} & \alpha_{12}\\ \alpha_{21} & \alpha_{22} \end{array} & \begin{array}{cc} \alpha_{13} & \alpha_{14}\\ \alpha_{23} & \alpha_{24} \end{array}\\ \begin{array}{cc} \alpha_{31} & \alpha_{32}\\ \alpha_{41} & \alpha_{42} \end{array} & \begin{array}{cc} \alpha_{33} & \alpha_{34}\\ \alpha_{43} & \alpha_{44} \end{array} \end{array}\right]$, $\alpha_{\mathrm{ii}}=1-\left(\overset{N}{\underset{j=1}{\sum }}{\left|S_{\mathrm{ij}}\right|}^{2}\right)$ and $\alpha_{\mathrm{ij}}=-\left(S_{\mathrm{ii}}^{*}S_{\mathrm{ij}}+S_{\mathrm{ji}}^{*}S_{\mathrm{ij}}\right)$.

**Figure 14:**
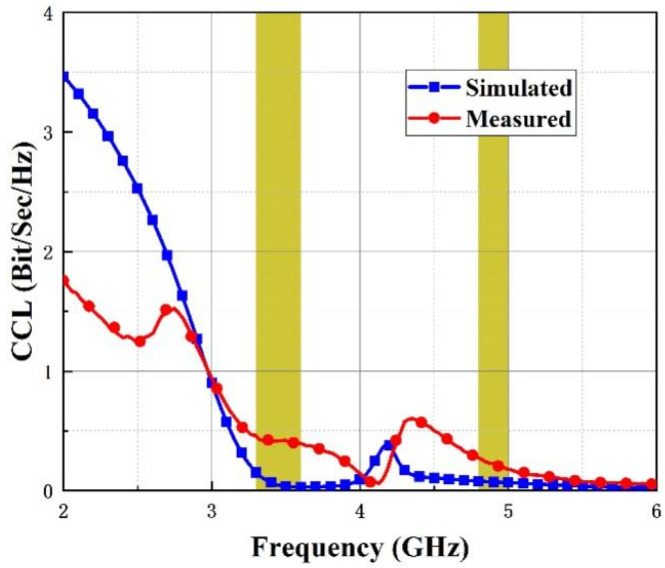
CCL of introduced four-port antenna.

Simulated and measured curves of the CCL have been presented in Figure 14, which illustrates that the CCL is below 0.4 bits/s/Hz and 0.5 bits/s/Hz at the lower and upper band, respectively.

**Figure 15:**
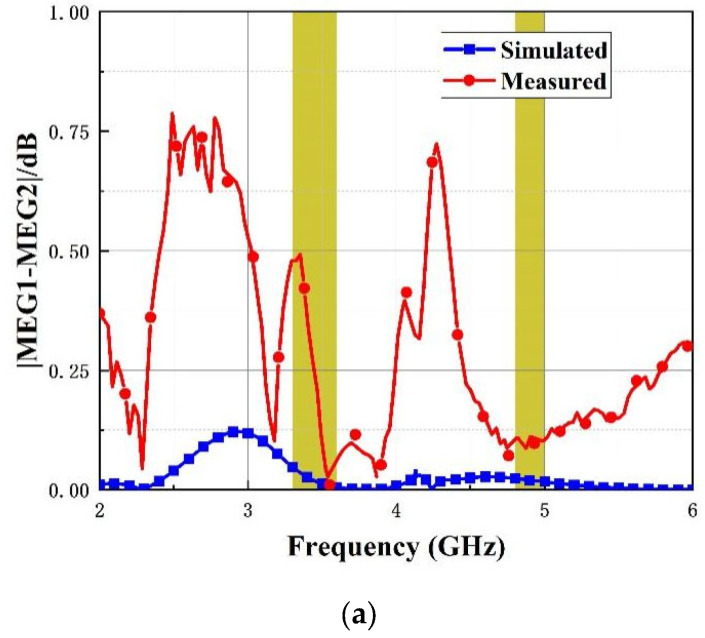

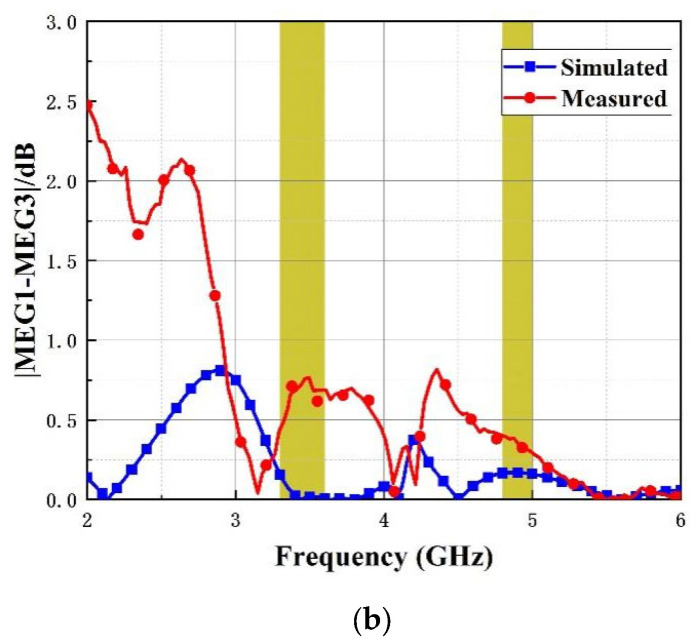
MEG of introduced four-port antenna. (**a**) |MEG1-MEG2| (**b**) |MEG1-MEG3|.

The mean effective gain (MEG) acts as another crucial parameter to evaluate the capability of MIMO antenna system. We can calculate the MEG by using the equation [21]:

(6) $${\mathrm{MEG}}_{i}=0.5\left[1-\sum_{J=1}^{N}{\left|S_{\mathrm{ij}}\right|}^{2}\right]$$

(7) $$\left|{\mathrm{MEG}}_{i}-{\mathrm{MEG}}_{j}\right|<3\mathrm{dB}$$

where N signifies the total number of the antenna ports. Generally, the difference between MEG_i_ should be less than 3 dB. The difference between the simulated and measured MEG values are plotted in Figure 15, showing that the value is less than 1 dB with the operating band.

### 4.4. TARC (Total Active Reflection Coefficient)

**Figure 16:**
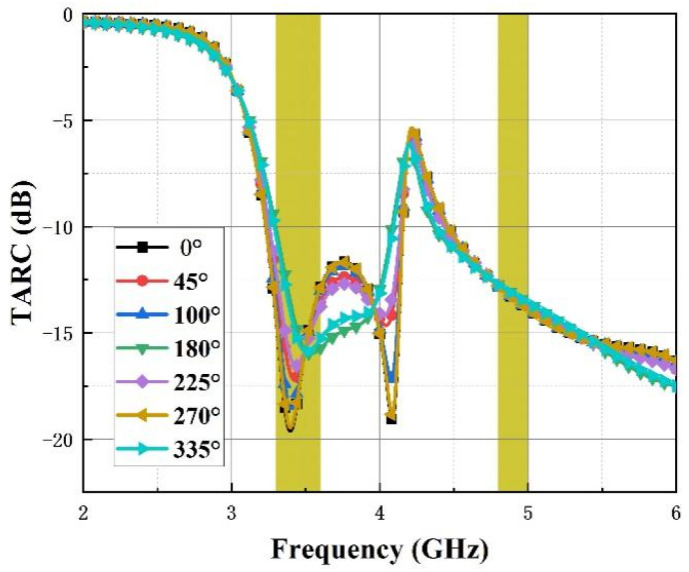
Antenna TARC.

The TARC is considered an important indicator and is commonly used to evaluate the performance of MIMO systems [22]. It can be calculated by Expression (8):

(8) $$\mathrm{TARC}=N^{-0.5}\sqrt{\sum_{i=1}^{N}{\left|\sum_{k=1}^{N}S_{\mathrm{ik}}e^{{j\theta }_{k-1}}\right|}^{2}}$$

where N represents the number of the antenna ports, S_ik_ represents the reflection coefficient from Port i to Port k and θ stands for a variable of phase ranging from 0° to 360°. As the proposed antenna has four ports, the value of N is four and there exists three variables of θ: θ_1_, θ_2_ and θ_3_. To simplify the analysis, seven values of θ_1_ (0, 45, 180, 225, 270, 335) are selected, keeping the values of θ_2_ and θ_3_ equal to 0. The curves of the TARC with the variation of θ_1_ are shown in Figure 16. It can be observed that the curves are below −10 dB within the functioning bands, which demonstrates that the antenna possesses an allowable performance for MIMO application.

### 4.5. Performance Comparison

In this paper, we make a comparison with previous antennae on the size, bandwidth, isolation, ECC, and the number of antenna elements as is demonstrated in Table 1. As can be seen, the illustrated antenna offers good capabilities over other antennae in terms of a wider bandwidth, a lower ECC, slightly compact dimensions and higher isolation. Moreover, the feeder method in this paper supports the concept of integration compared to the previous design, such as microstrip feeder.

## 5. Conclusions

A dual-band quad-element MIMO antenna operating at 3.45 GHz and 4.9 GHz bands has been designed for 5G applications. The antenna has achieved enhanced isolation by introducing a cross-shaped stub and modifying the ground structure which also works to extend the bandwidths. There is a desirable resemblance between the computed and tested results which shows the MIMO antenna is in possession of high isolation and a good impedance match throughout all the bands. Furthermore, the diversity performance such as gain, efficiency, ECC, DG, CCL, MEG and TARC has been investigated, the results of which confirm a good performance of the antenna. Based on these good results, the proposed antenna is suitable for 5G applications.

## Figures and Tables

**Table 1:** Performance comparison with other antennae.

Ref	Size (mm^2^)	Bandwidth (GHz)	Isolation (dB)	ECC	No. of Element	Feeder Method
[12]	2500	(2.25–3.15) 33.3%, (4.89–5.95) 19.6%	15	0.01	2	CPW
[15]	2162.25	(2.28–2.7)16.9%, (4.96–6.1) 20.6%	15	0.06	2	Microstrip feed
[17]	1587.2	(2.99–3.61) 18.8%, (4.53–4.92) 8.3%	16	0.002	2	Microstrip feed
[18]	1681	(3.471–3.529)1.7%, (5.678–5.721) 0.8%	18.4	0.08	4	Microstrip feed
[19]	1156	(3.35–3.75) 11.2%, (5.6–6.05) 7.7%	19	0.01	4	Microstrip feed
Prop.	4624	(3.28–4.15) 23.4%, (4.69–6.01) 24.7%	19	0.0025	4	CPW
